# Geographically linking population and facility surveys: methodological considerations

**DOI:** 10.1186/1478-7954-11-14

**Published:** 2013-08-08

**Authors:** Martha Priedeman Skiles, Clara R Burgert, Siân L Curtis, John Spencer

**Affiliations:** 1 Carolina Population Center, University of North Carolina at Chapel Hill, 206 W Franklin St, Chapel Hill, NC 27516, USA; 2 ICF International, 11785 Beltsville Drive, Suite 300, Calverton, MD 20705, USA

**Keywords:** Spatial linkage, DHS, SPA, Misclassification error

## Abstract

**Background:**

The relationship between health services and population outcomes is an important area of public health research that requires bringing together data on outcomes and the relevant service environment. Linking independent, existing datasets geographically is potentially an efficient approach; however, it raises a number of methodological issues which have not been extensively explored. This sensitivity analysis explores the potential misclassification error introduced when a sample rather than a census of health facilities is used and when household survey clusters are geographically displaced for confidentiality.

**Methods:**

Using the 2007 Rwanda Service Provision Assessment (RSPA) of all public health facilities and the 2007–2008 Rwanda Interim Demographic and Health Survey (RIDHS), five health facility samples and five household cluster displacements were created to simulate typical SPA samples and household cluster datasets. Facility datasets were matched with cluster datasets to create 36 paired datasets. Four geographic techniques were employed to link clusters with facilities in each paired dataset. The links between clusters and facilities were operationalized by creating health service variables from the RSPA and attaching them to linked RIDHS clusters. Comparisons between the original facility census and undisplaced clusters dataset with the multiple samples and displaced clusters datasets enabled measurement of error due to sampling and displacement.

**Results:**

Facility sampling produced larger misclassification errors than cluster displacement, underestimating access to services. Distance to the nearest facility was misclassified for over 50% of the clusters when directly linked, while linking to all facilities within an administrative boundary produced the lowest misclassification error. Measuring relative service environment produced equally poor results with over half of the clusters assigned to the incorrect quintile when linked with a sample of facilities and more than one-third misclassified due to displacement.

**Conclusions:**

At low levels of geographic disaggregation, linking independent facility samples and household clusters is not recommended. Linking facility census data with population data at the cluster level is possible, but misclassification errors associated with geographic displacement of clusters will bias estimates of relationships between service environment and health outcomes. The potential need to link facility and population-based data requires consideration when designing a facility survey.

## Background

Several studies have explored the relationships between health service availability and quality and health behaviors and outcomes [1-5]. Examining these relationships requires bringing together data on health outcomes with data on the relevant health service environment; interest in linking these two types of data is growing [6,7].

Household surveys such as the Demographic and Health Surveys (DHS) are a leading source of data on population health status and health care-seeking behavior, while health facility surveys are an increasingly accessible source of data on the availability and quality of health services. Separately, these data provide information on the population demand and service supply side environments, but researchers seeking to enrich analyses of population health data with an understanding of the service environment are offered limited insight into the relationship between the two. Establishing links between survey respondents and individual facilities has often relied on geographic proximity or respondent identification of facility(s) visited [8-15]. Another approach links household clusters to all facilities within a geographic area in an effort to portray survey respondents’ exposure to a service environment [16-19]. The increasing availability of geographic data in household and facility surveys presents an opportunity to link these types of data together using geo-spatial techniques. Geographic linking is particularly attractive because it has the potential to be an efficient approach that maximizes the use of existing data [20]. Linking these data sources, however, raises a number of methodological issues that have not been extensively explored.

In this paper we explore methodological issues in linking DHS household survey data with facility survey data from Service Provision Assessments (SPA). Both of these public data sources are collected by the MEASURE DHS project funded by the United States Agency for International Development (USAID) [21]. We focus on two particular methodological issues. First, SPA surveys typically use stratified samples of public and private facilities designed to provide a national picture of service delivery and statistically representative estimates for the first administrative level below the national level (e.g., province or region); they are not typically designed to provide statistically representative estimates at lower geographic levels [22]. Sampling is a cost-effective method of providing a national assessment of services but has implications for linking facility survey data to independent household survey data. Second, in a DHS the geographic locations of sampled clusters are displaced before public release to preserve confidentiality of respondents [23]. The potential effect of this displacement on data linking has been explored between survey clusters and population census data, but not between clusters and facilities [24].

The objective of this paper is to explore the potential misclassification error introduced when a sample rather than a census of health facilities is used and when household survey clusters are geographically displaced. We use a number of different approaches for linking household data with health facility data geographically to explore the extent to which measurement errors associated with facility sampling and cluster displacement vary across commonly used geographic linking methods.

This study was deemed exempt from review by the Office of Human Research Ethics at the University of North Carolina at Chapel Hill.

## Methods

### Data sources

Data from the Republic of Rwanda were used for this descriptive analysis. Rwanda was chosen as an example because geographic coordinates were available for Rwanda’s DHS and SPA surveys, the SPA was a census, and the surveys occurred within an 18-month window.

#### 2007–2008 Rwanda Interim Demographic and Health Survey (RIDHS)

The 2007–2008 RIDHS is a population-based household survey that used standard DHS questionnaires for family planning and maternal and child health. Data collection was carried out between December 2007 and April 2008 by the Rwanda National Institute of Statistics with technical assistance from the MEASURE DHS project [25].

The 2007–2008 RIDHS sample is a subsample of the 2005 Rwanda DHS. The 2005 DHS sample was a two-stage stratified area sample with 462 primary sampling units (PSUs) or clusters, drawn from a complete list of enumeration areas (EA) supplied by the 2002 General Population and Housing Census [26]. For the 2007–2008 RIDHS, 250 clusters from the 2005 DHS were selected and 30 households were randomly selected per cluster. The survey was successfully completed in 249 clusters; 185 clusters were located in rural areas. This analysis targets only rural areas due to challenges defining a health service environment in an urban setting with higher population and facility density, more private sector options, and more transportation potential.

The geographic locations of the 2005 Rwanda DHS clusters are represented by point coordinates located at the centroid of each cluster with no differentiation made for different size clusters. These points were collected using Global Positioning System (GPS) receivers and verified by MEASURE DHS [27]. Cluster GPS points are displaced up to 5 kilometers in rural areas with 1% of rural GPS points displaced up to 10 kilometers. Additionally, the data displacement was constrained by district boundaries. The displaced GPS data from the 2005 survey were used to create the 2007 GPS dataset. Three clusters were dropped from the dataset due to missing locations.

#### 2007 Rwanda Service Provision Assessment

The goal of the 2007 Rwanda Service Provision Assessment (RSPA) survey was to determine the extent to which facilities were prepared to provide high-priority maternal, child health, and HIV/AIDS services. Data were collected from a sample of providers and clients at each facility, covering family planning, antenatal care, HIV/AIDS, sexually transmitted infections, and child curative care services [28].

A total of 555 facilities managed by the government, non-governmental organizations, and communities were sampled for the survey, and 538 were successfully interviewed. Sampled facilities included 42 hospitals; 389 health centers and polyclinics; and 107 dispensaries, health posts, and clinics. The sample included all public health facilities, all private facilities that had five or more staff assigned or employed by the facility at the time of listing, and one-third of private facilities that had three to four health workers. Private facilities with one or two staff were excluded from the survey.

The geographic locations of the SPA facilities were collected during the survey using GPS receivers and verified using data on health facility locations from the Rwanda Ministry of Health. These geographic facility data were not displaced. Fourteen of the 538 facilities were dropped from the dataset due to missing geographic data.

#### Geographic data – roads, shape files

Additional geographic data used in the analysis includes administrative polygons and national road network data. The administrative polygons, from the Rwanda Ministry of Health, reflect administrative boundaries established in 2006. The road network was created from Open Street Map [29] and cleaned to assure continuous road segments.

### Linking methods

**Figure 1:**
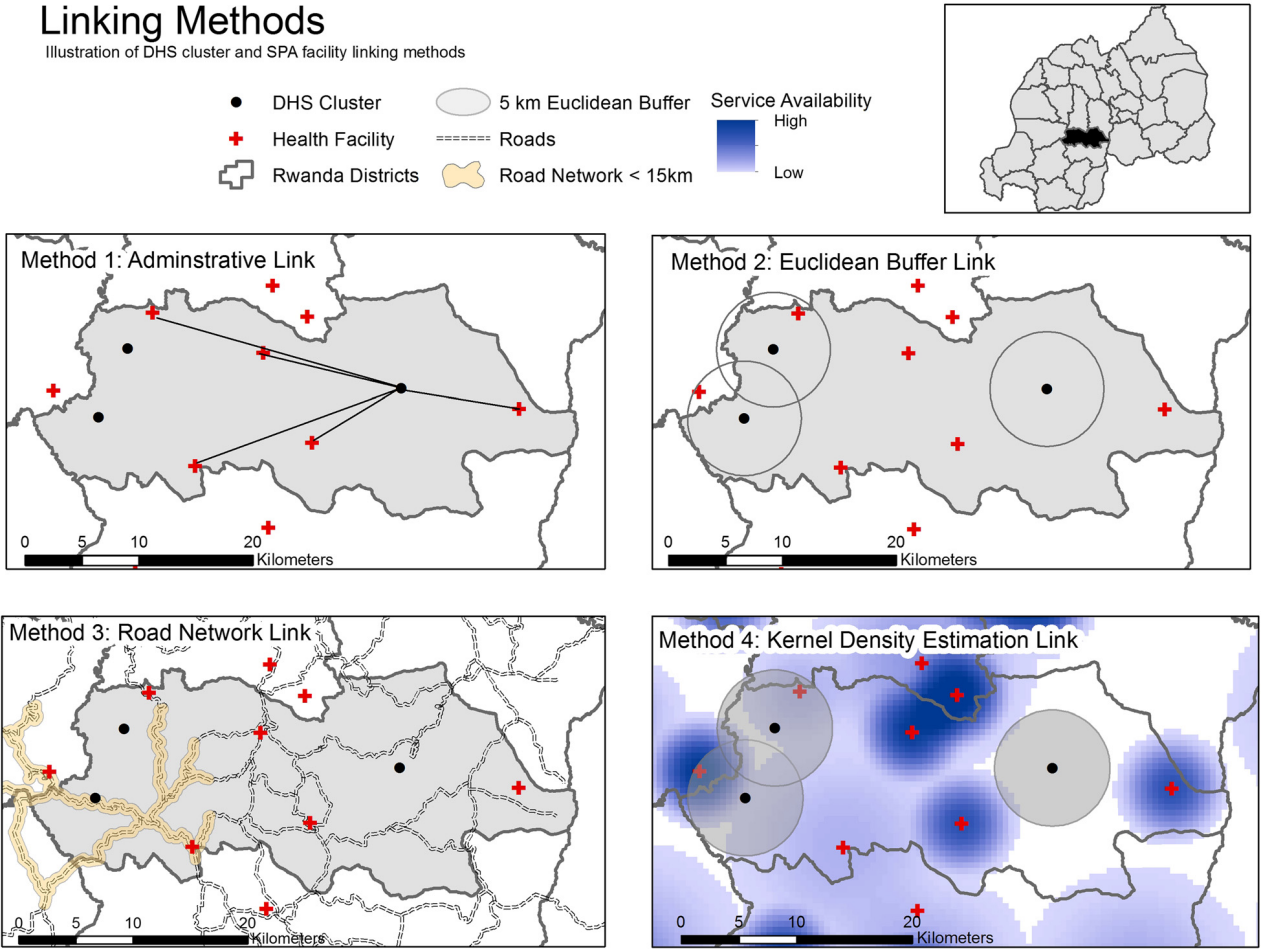
Illustration of DHS cluster and SPA facility linking methods.

We applied three commonly used methods for directly linking clusters with facilities: administrative boundary link, Euclidean buffer link, and road network link (Figure 1). A fourth method, kernel density estimation (KDE), was used to approximate the relative influence by one or more facilities on a cluster [30].

#### Administrative boundary link

DHS clusters were linked with health facilities located within the same administrative polygon, in this case the district.

#### Euclidean buffer link

A 5 kilometer (km) Euclidean buffer was centered on each DHS cluster to approximate a one-hour walking distance from cluster centroid to facility. The cluster was then linked to each health facility located within the buffer, without consideration of administrative borders.

#### Road network link

The distance along a road from a cluster to a facility is the parameter that defines the link. This distance value is calculated by summing up the distance from a cluster to the nearest road within 5 km, the distance along the road, and the distance from the facility to the road again within 5 km. All summed distances less than 15 km were retained as a link.

#### Kernel density estimation link

KDE is a technique employed to distribute a value associated with a discrete point across a plane or continuous surface. In the case of health facilities, one assumes that a facility serves a geographic catchment area, yet the draw on the population to those services likely decreases as distance from the facility increases. Likewise, the draw of the facility varies by facility type, size, and availability of services. With KDE, one can incorporate facility characteristics and distance decay when estimating the potential draw a facility may have on a population cluster. The KDE link requires user-defined kernel size, density variable to determine the probability density distribution across the kernel, and grid size. The kernel size was chosen to reflect preference for higher-level facilities: 10 km for hospitals, 5 km for health centers, and 2.5 km for dispensaries [31]. Two density variables, family planning (FP) and HIV voluntary counseling and testing (VCT) readiness scores, were used with a Gaussian distribution. The grid cell size was set to 500 meters. The KDE for each facility type was created separately and then summed within each grid cell using the Map Algebra Raster calculator tool to create the KDE total layer. Because the DHS cluster GPS is taken at the centroid and we know the cluster population is dispersed over a surface area, we generated an average KDE value for each cluster by superimposing 5 km Euclidean buffers around each DHS cluster. Using the spatial analyst tools in ArcGIS, we averaged the KDE weights for the total 5 km surface for each cluster.

All geographic linking was conducted in ArcGIS v10 (Redlands, CA) using spatial analyst and network analyst extensions; linked datasets were exported to Stata SE v12 (College Station, TX) for analysis.

### Health facility samples and cluster displacement

In total, 36 datasets were constructed for each linking method: one census/undisplaced linked master, five facility samples linked with the undisplaced clusters, five cluster displacements linked with the facility census, and 25 facility samples/cluster displacements. The master dataset includes the original 185 rural DHS clusters linked to the full SPA facility census file.

To explore the implications of sampling in SPA surveys, five facility samples of 260 facilities each were drawn from the master SPA census file to simulate a typical SPA sample dataset. Each facility sample included all 42 hospitals and all 23 large private facilities from the master file, plus 195 additional lower-level facilities selected by stratified sampling according to facility type with a proportional allocation by type and region (implicit). The original DHS dataset was then linked to each of these SPA sample datasets, creating five datasets for the sample and undisplaced analysis.

To examine the potential error introduced by cluster displacement, the GPS locations of the 185 DHS clusters were displaced five times using the standard DHS displacement algorithm, creating five comparative DHS datasets. The DHS cluster locations in the original dataset were already displaced, but for the purposes of this analysis we consider those as the “true” locations because our focus is on the relative difference in the results when cluster locations are displaced. The SPA facility census data were then linked to each of these displaced DHS datasets, creating five datasets for the census/displaced analysis.

Lastly, to explore the combined effect of SPA sampling and cluster displacement, we linked each of the five facility sample datasets with each of the five displaced cluster datasets to create 25 facility sample and cluster displaced datasets.

### Health service environment measures

The links between the DHS clusters and health facilities were operationalized by creating health service variables from the SPA facility characteristics to attach to the linked DHS clusters. For the three direct linking methods we created the following health service environment variables: distance to nearest health facility; number of health facilities linked to the cluster; type of linked facilities; FP methods available in at least one facility linked to the cluster; and HIV services available in at least one facility linked to the cluster. Each contraceptive method was coded as available if the facility reported providing that method and if the interviewer confirmed that the method was in stock on the day of the interview. The HIV services observed included: VCT; basic prevention of mother-to-child transmission (PMTCT) of HIV, which includes VCT, infant feeding counseling, FP counseling, and antiretroviral (ARV) prophylaxis for pregnant women; and antiretroviral treatment (ART) for any HIV-positive clients. In the RSPA, data on HIV services were collected from multiple clinics or units within larger facilities. In this analysis, a facility is counted as offering the service if at least one unit reported offering the service in-house.

For the KDE link, we created two composite indices to measure FP readiness and VCT readiness and assigned the mean scores across linked facilities for each cluster. For FP services, we adopted the index created by Wang and colleagues [32]. Fifteen dichotomous variables measuring four dimensions of FP services were summed for each facility. The four dimensions of care included: FP counseling, infection control, pelvic examination, and management practices.

An analogous measure for VCT service readiness was created based on service readiness indicators proposed by the World Health Organization (WHO) and USAID [33]. Seven dichotomous variables sum to the composite index and measure counseling and testing, condom availability, and management practices.

### Analysis

For this descriptive sensitivity analysis we first compared the distribution of key variables in the master dataset (census/undisplaced) with the corresponding distributions in the sample and displaced linked datasets and examined the percent disagreement for each comparison. Logistic regression models assessed the association between health service environment, measured as access to a facility within 5 km, and use of modern contraception. Models were run for the master dataset and the facility sample/cluster undisplaced datasets within a 5 km buffer.

To explore the extent to which variables representing relative service environment are affected by facility sampling and cluster displacement, we created relative measures of FP and VCT readiness. For the three direct linking methods, clusters were divided into quintiles based on their mean readiness scores and assigned a value representing the quintile placement in that dataset (1=lowest quintile to 5 = highest quintile). For the KDE linking, we created quintiles from the KDE values for the readiness scores for each dataset. The quintile boundaries vary across datasets reflecting variation in the distribution of the scores across datasets. Comparisons were made between quintiles from the master dataset and the facility sample/cluster displaced datasets. Logistic regression models assessed the association between these relative health service environments and the use of modern contraception.

## Results

### Clusters to facilities: direct links

Table 1 presents the distribution of linked clusters across health service variables for the master dataset compared to the facility samples and displaced clusters datasets. Comparing first the master dataset across linking methods, we find the distance to the closest facility is similar; although links to more facilities, more types of health facilities, and more FP methods and HIV services are found when linking by administrative boundary compared to the 5 km buffer. Results from the road network link were similar to the buffer link (data not shown). These differences reflect the size of the geographic area encompassed by each linking method.

**Table 1:** Number (percent) of DHS clusters linked to health facilities by linking method and facility characteristics, comparing the census/undisplaced data with facility samples and displaced clusters (N=185 clusters)

	**Administrative boundary**						**Euclidean buffer (5 km)**					
	**Census/ undisplaced**		**Facility samples (5)**		**Displaced clusters (5)**		**Census/ undisplaced**		**Facility samples (5)**		**Displaced clusters (5)**	
	**No.**	**(%)**	**Range**	**(%)**	**Range**	**(%)**	**No.**	**(%)**	**Range**	**(%)**	**Range**	**(%)**
**Distance to closest facility**												
< 2.5 km	66	(35.7)	(22–39)	(11.9–21.1)	(60–74)	(32.4–40.0)	68	(36.8)	(22–40)	(11.9–21.6)	(62–77)	(33.5–41.6)
2.5 – 5 km	95	(51.4)	(59–67)	(31.9–36.2)	(81–101)	(43.8–54.6)	100	(54.1)	(64–77)	(34.6–41.6)	(87–107)	(47.0–57.8)
5.1–10 km	24	(13.0)	(59–80)	(31.9–43.2)	(23–30)	(12.4–16.2)	0	(0.0)	(0–0)	(0.0–0.0)	(0–0)	(0.0–0.0)
**Number of facilities**												
0	0	(0.0)	(0–0)	(0.0–0.0)	(0–0)	(0.0–0.0)	17	(9.2)	(77–91)	(41.6–49.2)	(16–23)	(8.6–12.4)
1 – 3	0	(0.0)	(0–7)	(0.0–3.8)	(0–0)	(0.0–0.0)	153	(82.7)	(92–104)	(49.7–56.2)	(142–157)	(76.8–84.9)
4 or more	185	(100)	(178–185)	(96.2–100)	(185–185)	(100–100)	15	(8.1)	(1–4)	(0.5–2.2)	(11–20)	(5.9–10.8)
**Type of facilities**												
Hospital	172	(93.0)	(172–172)	(93.0–93.0)	(172–172)	(93.0–93.0)	25	(13.5)	(25–25)	(13.5–13.5)	(22–27)	(11.9–14.6)
Health center	185	(100)	(185–185)	(100–100)	(185–185)	(100–100)	166	(89.7)	(74–88)	(40.0–47.6)	(157–166)	(84.9–89.7)
Health post	137	(74.1)	(114–126)	(61.6–68.1)	(137–137)	(74.1–74.1)	25	(13.5)	(18–20)	(9.7–10.8)	(27–31)	(14.6–16.8)
**FP methods available**												
Pill	178	(96.2)	(170–178)	(91.9–96.2)	(178–178)	(96.2–96.2)	128	(69.2)	(52–68)	(28.1–36.8)	(121–126)	(65.4–68.1)
Injectable	185	(100)	(170–178)	(91.9–96.2)	(185–185)	(100–100)	121	(65.4)	(46–67)	(24.9–36.2)	(113–121)	(61.1–65.4)
Implant	139	(75.1)	(94–114)	(50.8–61.6)	(139–139)	(75.1–75.1)	49	(26.5)	(16–20)	(8.6–10.8)	(43–50)	(23.2–27.0)
**HIV services available**												
VCT	185	(100)	(167–185)	(90.3–100)	(185–185)	(100–100)	138	(74.6)	(52–75)	(28.1–40.5)	(126–137)	(68.1–74.1)
PMTCT	185	(100)	(163–185)	(88.1–100)	(185–185)	(100–100)	107	(57.8)	(40–57)	(21.6–30.8)	(98–106)	(53.0–57.3)
ART	185	(100)	(178–185)	(96.2–100)	(185–185)	(100–100)	86	(46.5)	(37–50)	(20.0–27.0)	(83–93)	(44.9–50.3)

PMTCT includes VCT, counseling for infant feeding and family planning, and ART for pregnant women.

Next we compared the distributions of each variable between the master dataset and the five facility sample linked datasets. The facility sample datasets systematically underestimate the percentage of clusters that are within 5 km of a health facility, underestimate the number and type of linked facilities within 5 km, and underestimate the percentage of clusters that are linked to a facility providing each contraceptive method and each HIV service compared to the facility census dataset. These differences are smaller when linked by the geographically larger administrative unit.

Lastly, comparing the change in distribution of variables when linking with displaced clusters, the differences are found to be minimal. With the administrative boundary linking method, only the distance to the closest health facility is affected by the cluster displacement. This is because clusters are not displaced across district boundaries. Some variability is introduced by the cluster displacement when linking within the 5 km buffer, but the differences are relatively small and not systematic.

Table 2 shows the percent of clusters misclassified when compared to the master dataset, quantifying the potential measurement error introduced when linking DHS with a sample rather than a census of facilities or displacing DHS clusters. Linking by administrative boundary, distance to the closest facility was misclassified for 43-51% of clusters in the facility sample linked datasets compared to the master dataset and for 35-43% of the clusters in the cluster displaced linked datasets. In these descriptive analyses, sampling facilities generally results in larger misclassification error than cluster displacement, and linking to all facilities within the same administrative boundary results in the least amount of misclassification error.

**Table 2:** Percent of clusters with links misclassified when moving from a facility census to a facility sample with undisplaced and displaced clusters, by linking method (N=185 clusters)

	**Facility samples with undisplaced clusters**	**Facility census with displaced clusters**	**Facility samples with displaced clusters**
**Error due to:**	**(Sampling)**	**(Displacement)**	**(Sampling/displaced)**
**Administrative boundary**			
**Distance to closest facility**	(43.2–50.8)	(35.1–42.7)	(58.4–71.9)
**Number of linked facilities**	(5.9–12.4)	(0.0–0.0)	(5.9–12.4)
**Type of linked facilities**			
Hospital	(0.0–0.0)	(0.0–0.0)	(0.0–0.0)
Health center	(0.0–0.0)	(0.0–0.0)	(0.0–0.0)
Health post	(5.9–12.4)	(0.0–0.0)	(5.9–12.4)
**FP methods available by linked facilities**			
Pill	(0.0–4.3)	(0.0–0.0)	(0.0–4.3)
Injectable	(3.8–8.1)	(0.0–0.0)	(3.8–8.1)
Implant	(13.5–24.3)	(0.0–0.0)	(13.5–24.3)
**HIV services available by linked facilities**			
VCT	(0.0–9.7)	(0.0–0.0)	(0.0–9.7)
PMTCT	(0.0–11.9)	(0.0–0.0)	(0.0–11.9)
ART	(0.0–3.8)	(0.0–0.0)	(0.0–3.8)
**Euclidean buffer (5 km)**			
**Distance to closest facility**	(38.9–45.9)	(33.5–43.2)	(56.2–67.6)
**Number of linked facilities**	(49.7–59.5)	(21.6–28.6)	(49.2–66.5)
**Type of linked facilities**			
Hospital	(0.0–0.0)	(5.9–9.2)	(5.9–9.2)
Health center	(42.2–49.7)	(7.0–12.4)	(42.7–57.8)
Health post	(2.7–3.8)	(4.9–7.6)	(5.4–9.7)
**FP methods available by linked facilities**			
Pill	(32.4–41.1)	(7.6–15.1)	(34.1–48.6)
Injectable	(29.2–40.5)	(9.7–14.6)	(31.9–45.9)
Implant	(15.7–17.8)	(7.0–16.2)	(14.6–23.2)
**HIV services available by linked facilities**			
VCT	(34.1–46.5)	(17.3–21.1)	(37.8–52.4)
PMTCT	(27.0–36.2)	(16.2–21.1)	(32.4–46.5)
ART	(19.5–26.5)	(15.1–24.9)	(24.9–39.5)

PMTCT includes VCT, counseling for infant feeding and family planning, and ART for pregnant women.

Table 3 illustrates the potential bias introduced to a regression analysis by facility sampling in a 5 km buffer link. The measurement error in this simple non-linear regression biases both the direction and magnitude of the marginal effect. Controlling for some common predictors of contraceptive use reduces some of the noise in the marginal effect but does not eliminate the effect of misclassification. In a basic linear regression with non-differential misclassification, attenuated effects are predicted [34]; however, the direction of bias in a non-linear regression with differential misclassification is unpredictable [35]. Similar results were found when isolating the effects of cluster displacement (data not shown).

**Table 3:** Marginal effect at the mean for health facility access within 5 km and individual use of modern contraception, modeled for different facility datasets linked to undisplaced clusters

	**Model: census**	**Model: sample 1**	**Model: sample 2**	**Model: sample 3**	**Model: sample 4**	**Model: sample 5**
	**ME (se)**	**ME (se)**	**ME (se)**	**ME (se)**	**ME (se)**	**ME (se)**
Facility ≤ 5km	0.024	0.033***	−0.009	0.035***	0.004	−0.008
	(0.017)	(0.010)	(0.010)	(0.010)	(0.010)	(0.010)
Facility ≤ 5km, with covariates^1^	0.014*	0.013**	−0.003	0.014**	−0.001	−0.004
	(0.007)	(0.005)	(0.004)	(0.005)	(0.004)	(0.004)

^1^ Controlling for marital status, mother’s education, parity, desire to space/limit children, wealth.

* p<0.05, **p<0.01, *** p<0.001.

### Clusters to facilities: weighted links

**Figure 2:**
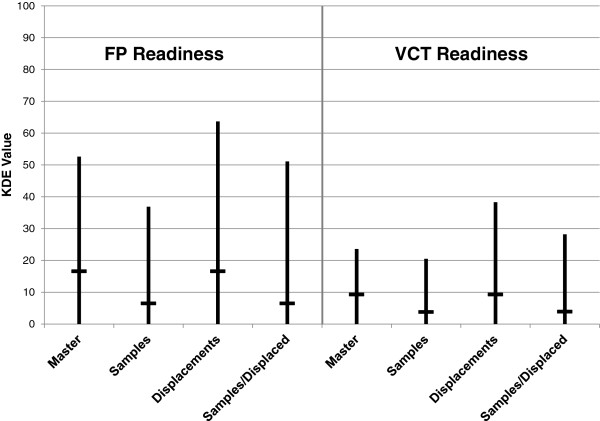
Mean FP and VCT readiness scores for DHS clusters using KDE linking methods (N=185 clusters).

**Figure 3:**
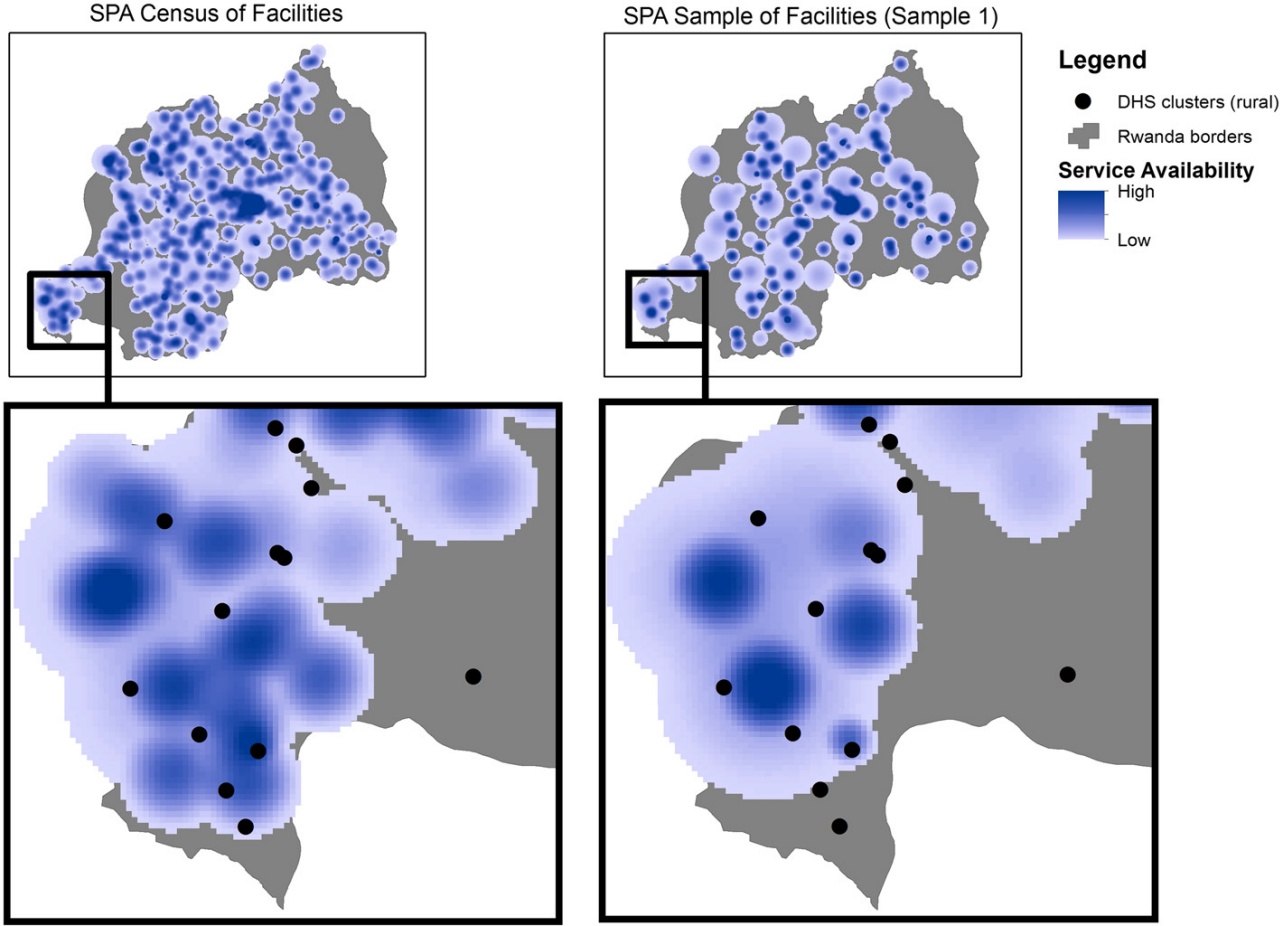
KDE to compare a census and a sample of facilities on VCT readiness.

In the master dataset, the mean KDE values for the FP and VCT readiness scores are 16.6 and 9.3, respectively (Figure 2). Cluster displacement introduces some variability into the range of values (vertical bar) but the means remain the same (horizontal bar). Sampling, however, greatly reduces the mean KDE values for both readiness scores, in effect underestimating access to facilities with adequate FP or VCT services. Figure 3, which maps the VCT readiness score for the master dataset and for one sample linked dataset, illustrates the substantial effect of sampling on estimated access to adequate VCT services by clusters.

So far, we have focused on the absolute values of variables that describe the health service environment around a cluster. What may be meaningful and potentially more robust is the relative service environment. Using relative service readiness measures and comparing the master dataset to the samples, we found a minimum of 36% of the clusters assigned to an incorrect FP readiness quintile when administratively linked with a sample of facilities and 60% when using KDE methods (Table 4). When linking with displaced clusters, more than one-third were misclassified regardless of linking method. In most of the combined sample/displaced datasets, over half of the clusters were classified into the incorrect quintile relative to the census/undisplaced master dataset; particularly for the VCT readiness score. Regression results using the master dataset show an increasing effect on contraceptive use as the relative FP readiness score increases (Table 5). However as seen earlier, the potential bias introduced by the measurement error from facility sampling influences the marginal effects both in magnitude and direction.

**Table 4:** Percent of clusters with readiness scores misclassified by quintile when moving from a facility census to a facility sample with undisplaced and displaced clusters, by linking method (N=185 clusters)

**Readiness measure**	**Facility samples with undisplaced clusters**	**Facility census with displaced clusters**	**Facility samples with displaced clusters**
	**(Samples)**	**(Displacements)**	**(Samples/Displaced)**
**Family planning**			
Administrative boundary	(35.7–62.2)	(0.0–0.0)	(35.7–62.2)
Euclidean buffer (5 km)	(59.5–65.9)	(36.8–48.1)	(61.6–78.4)
KDE	(60.0–68.6)	(34.6–39.5)	(58.9–72.4)
**VCT**			
Administrative boundary	(38.9–78.4)	(0.0–0.0)	(38.9–78.4)
Euclidean buffer (5 km)	(61.6–70.3)	(38.4–41.1)	(60.5–78.4)
KDE	(61.1–69.2)	(36.2–44.9)	(60.5–74.6)

**Table 5:** Marginal effect at the mean for family planning readiness score and individual use of modern contraception, modeled for different datasets linked with KDE

	**Model: census**	**Model: sample 1**	**Model: sample 2**	**Model: sample 3**	**Model: sample 4**	**Model: sample 5**
	**ME (se)**	**ME (se)**	**ME (se)**	**ME (se)**	**ME (se)**	**ME (se)**
**FP readiness score (ref=lowest score):**						
Low score	−0.027	−0.024	0.008	−0.019	−0.023	−0.009
	(0.015)	(0.015)	(0.016)	(0.016)	(0.015)	(0.015)
Middle score	0.020	0.015	−0.027	0.005	−0.014	−0.020
	(0.017)	(0.016)	(0.015)	(0.016)	(0.015)	(0.015)
Higher score	0.038*	0.025	−0.018	0.020	0.034*	−0.003
	(0.017)	(0.017)	(0.015)	(0.017)	(0.017)	(0.015)
Highest score	0.046**	0.028	0.009	0.051**	0.012	0.001
	(0.018)	(0.017)	(0.016)	(0.018)	(0.016)	(0.016)
**FP readiness score with covariates**^**1**^						
Low score	−0.007	−0.010	0.000	−0.008	−0.011	−0.008
	(0.007)	(0.007)	(0.007)	(0.007)	(0.006)	(0.006)
Middle score	0.009	0.007	−0.006	0.003	−0.001	−0.008
	(0.008)	(0.007)	(0.007)	(0.007)	(0.007)	(0.006)
Higher score	0.014	0.013	−0.008	0.010	0.011	−0.001
	(0.008)	(0.008)	(0.006)	(0.008)	(0.008)	(0.007)
Highest score	0.018*	0.012	0.003	0.021*	0.003	−0.004
	(0.008)	(0.008)	(0.007)	(0.008)	(0.007)	(0.007)

^1^ Controlling for marital status, mother’s education, parity, desire to space/limit children, wealth.

* p<0.05, **p<0.01, *** p<0.001.

### Populations to facilities: direct links

The final analyses explore the link between clusters and facilities from the perspective of the characteristics of the cluster populations. We compared the socio-demographic characteristics of women from clusters linked and not linked to a facility within the 5 km buffer when using the master dataset with those of women from clusters linked and not linked to a facility from the facility samples datasets. Facility sampling, as seen earlier, contributes the larger measurement error and the 5 km buffer is the most geographically restrictive link, hence this comparison offers the likely “worst case” scenario for selection effects. One might expect women from more remote location reporting less education, larger families, and increased poverty; the comparison between the linked and unlinked women in the master dataset suggest this (Table 6). Differences between linked/unlinked women in the master dataset are blurred, however, when linking to a facility sample because formerly linked women are misclassified as unlinked.

**Table 6:** Percent of women by socio-demographic characteristics from clusters linked and unlinked to a census and a sample of facilities within a 5 km Euclidean buffer

	**Facility census**		**Facility samples**	
**Characteristics**	**Linked**	**Unlinked**	**Linked**	**Unlinked**
No education	23.0	28.8	(22.0 – 23.7)	(23.3 – 25.2)
Wealth quintiles				
Poorest	24.3	21.8	(21.4 – 23.8)	(24.4 – 26.8)
Least poor	20.4	13.8	(20.0 – 23.2)	(16.0 – 19.6)
Parity				
0 Births	34.4	28.6	(33.0 – 34.9)	(32.8 – 34.8)
5 or more Births	24.9	30.3	(24.7 – 25.8)	(25.0 – 26.5)
Knowledge of the pill	88.9	83.1	(88.1 – 90.7)	(86.0 – 88.7)
Use of modern contraception^1^	15.8	13.3	(15.2 – 17.3)	(13.8 – 16.0)
Number of clusters	168	17	(94 – 108)	(77 – 91)
Number of women	4812	472	(2706 – 3075)	(2209 – 2573)

^1^Current contraceptive use among married women only.

The specific facility used by a woman for FP services is not available in DHS data, although linking women with the facility they used is often of substantive interest to researchers. Table 7 reports the percentage of women who were linked to a facility of the same type that they reported using for contraception and the percentage of women who were linked to a facility providing the contraceptive method they reported using. This provides an upper bound on the likelihood that a woman was linked to a facility she used for FP. For example, 81 women reported receiving their contraceptive method from a hospital; 7% of these women were linked with a hospital as their closest facility, this increased to a 96% match rate when the women were linked to all facilities within the administrative boundary.

**Table 7:** Percent of women currently using modern contraceptives who are linked to a facility that matches the reported source of method and the type of method used

**Type of link:**	**Administrative boundary**			**Euclidean buffer (5 km)**			
	**Facility census**	**Facility samples mean**	**Facility samples (range)**	**Facility census**	**Facility samples mean**	**Facility samples (range)**	**Number of women**
**Linked to closest facility**							
Last source of contraception							
Hospital	7.4	25.2	(21.0–30.9)	7.4	15.3	(11.1–17.3)	81
Health center	91.3	73.5	(68.5–76.3)	84.3	41.5	(36.9–45.4)	645
Dispensary	2.4	8.8	(4.9–14.6)	2.4	0.5	(0.0–2.4)	41
Current method							
Pill	59.1	48.5	(40.9–58.0)	56.8	27.4	(20.5–34.7)	176
Implant	25.5	11.4	(3.9–19.6)	21.6	6.3	(2.0–15.7)	51
**Linked to all facilities in service area**^**1**^							
Last source of contraception							
Hospital	96.3	96.3	(96.3–96.3)	28.4	28.4	(28.4–28.4)	81
Health center	100.0	100.0	(100.0–100.0)	92.7	45.5	(40.5–49.5)	645
Dispensary	80.5	72.7	(70.7–75.6)	9.8	3.9	(2.4–7.3)	41
Current method							
Pill	92.6	89.9	(85.8–92.6)	67.0	30.0	(22.7–39.2)	176
Implant	72.5	47.8	(39.2–72.5)	25.5	10.2	(3.9–15.7)	51

^1^Service area includes all facilities linked via boundary or buffer.

Linking to all facilities within an administrative boundary performs best in terms of linking women to a facility of the same type where they obtained their method, or to a facility that provides their method. Linking a cluster to the closest facility reduces the match rate across all variables, datasets, and linking methods. Linking with a sample of facilities rather than the census typically reduces the match rate; the matched rate is halved when using the 5 km buffer linked datasets. Notably, common compared to rare occurrences are more likely represented in linked data as demonstrated by the higher match rate for women reporting use of a health center versus a dispensary or using the pill compared to an implant.

## Discussion

Linking together data on health service environment and data on population health behaviors and outcomes is of considerable public health interest. Linking existing public datasets is particularly attractive as it has the potential to be an efficient approach to expanding our knowledge of the relationships between health services and health outcomes. The increasing availability of geo-referenced datasets provides great potential for expanded research, but our analysis of spatially linking two important global data sources – the DHS and the SPA - demonstrates that methodological challenges remain before realizing this potential.

### Effects of facility sampling

Our results show that when linking to a population survey, the facility sampling typically used in SPA surveys leads to substantial underestimation of the adequacy of the health service environment and substantial misclassification error for individual clusters. This is not surprising given that many commonly used health service environment variables are functions of the number of health facilities a cluster is linked to, and sampling will reduce those links. However, substantial misclassification error was also found when considering variables measuring the relative service environment, which we had expected to be less sensitive to the number of linked facilities. This finding reflects the fact that SPA survey samples are designed to be statistically representative at the first stratum domain (typically region or province), with a known, acceptable level of sampling error. This sampling is not designed to provide statistically representative estimates for small geographic areas such as those around a DHS cluster. The misclassification error introduced by sampling is likely to be differential; remotely located clusters are less likely to be linked to multiple facilities from the facility census and hence more likely to be misclassified as not being linked to a facility when facility samples are used in linking. This differential misclassification of access to services may bias estimates and produce spurious relationships between available health services and contraceptive use [34].

### Effects of geographic displacement

The geographic displacement of DHS cluster data is done to protect the confidentiality of respondents. The tension between confidentiality and accuracy is an ongoing debate [36]. Many Institutional Review Boards require steps to be taken to protect confidentiality and minimize deductive disclosure risks even with data that might not be considered highly sensitive; often this takes the form of modifications of coordinate data, such as displacement. The DHS coordinate displacement causes no additional error when linking to all facilities within an administrative boundary; however non-trivial misclassification at the individual cluster level is evident when lower-level geographic links are performed, particularly if attempting to link to the closest facility. The displacement errors appear to be largely random, likely due to the random nature of the cluster displacement; hence the descriptive analyses are still informative. However, the cluster-level misclassification in the regression models led to unpredictable, biased estimates when relating the health service environment to health outcomes at the individual level.

### Performance of linking methods

We explored different commonly used approaches for linking health facility data to household clusters. The differences in results between the tested linking methods largely reflect the different geographic boundaries associated with each method. The administrative boundary method links clusters with the most facilities and is the least affected by the facility sample and cluster displacement issues. However, it also produces relatively little variation in several of the health service environment variables, so it may not be very useful for analysis, and it may not represent a meaningful service environment for many respondents. The 5 km buffer and 15 km road network methods aim to address those concerns but are more affected by cluster displacement and sampling since they represent smaller geographic areas. Our results also show that linking to the closest facility performs poorly in terms of linking respondents with a facility of the same type or providing specific services that they report using. For analyses that conceptually depend on linking respondents to the facility they use, linking to the closest facility is inappropriate even when using a facility census and undisplaced clusters. Ultimately, the choice of linking method should be driven by the specific research questions and underlying theory.

Kernel density estimation represents an alternative approach to attaching health service environment characteristics to DHS clusters in a manner that takes into account multiple service delivery points with finite service resources in a relevant geographic space. However, this more sophisticated spatial analytic method did not appear to perform any better than the direct linking methods in terms of relative misclassification at the cluster level.

Previous analyses of the relationship between health services and health outcomes have relied on a number of different methods and data sources. One approach is to link household survey data to a detailed facility census, either at a national level or for a smaller geographic area in which the household survey was conducted [8,9,12]. This approach has the advantage of providing a complete picture of the service environment around a population and in many ways represents the ideal situation for spatial linking. However, detailed data collection for a census of facilities is very expensive and often not feasible for large geographic areas.

A second approach relies on a census of facilities located in the household survey cluster or EA and located in one or two concentric rings of neighboring EAs, plus all large facilities irrespective of location [6,16,37,38]. This approach provides a facility census around the household survey cluster for linked analysis. Additionally, it allows the facility data to be weighted based on the known selection probabilities of all the EAs, thereby providing representative national facility estimates [39]. This method attempts to balance competing objectives of facility surveys to provide data that can be linked to population surveys and also to provide representative estimates of facility indicators, while limiting the geographic area in which an expensive facility census is conducted.

Another common method is represented by the DHS service availability module where data on the health service environment for a DHS cluster are represented by the closest facility of each type in a defined geographic area [5,7,40-42]. This approach can be designed to give a picture of the health service environment around a cluster for linked analysis and provides representative estimates of population-based access indicators such as the percentage of the population living within a given distance of a health facility. However, additional data collection is required to determine the selection probabilities in order to obtain representative national estimates of facility characteristics, which is the primary objective of surveys like the SPA. Moreover, the focus on nearest facility (or nearest facility of each type) limits its application for other purposes that require a more comprehensive view of the service environment, as this study has illustrated.

Yet another method is to collect data from individual women and community key informants on health facilities used and conduct a survey or census of the facilities named by the surveyed population [19,43]. This method provides facility data for the choice set of facilities used by a community and allows individual women to be linked to the actual facility used, which may be important conceptually for some types of linked analyses. Yet again, this method does not provide representative national or subnational estimates of facility indicators due to selection bias.

The linking methods applied in this study, while commonly used, are relatively simple and do not make use of additional information that may be available to improve the precision of high spatial resolution estimates from facility surveys. New country efforts to create master facility lists will provide comprehensive sampling frames for health facilities at the EA level. This may enable modeling the systematic misclassification error of facility sampling, leading to better control of this error in regression analysis. More sophisticated analytic methods, such as using master facility lists to calibrate facility sample data for small area estimation, a method demonstrated by researchers linking population-based survey data with census data and facility data with population census data, warrants further study [9,15,32,44].

### Study limitations

Our study was conducted in only one purposively selected setting, Rwanda. Given the focus on methodological issues rather than substantive ones, our findings should be generalizable to other countries collecting DHS and SPA data because they represent the potential effects of standard SPA sampling and DHS cluster displacement methods. The sample size for our simulated facility samples was designed to provide a 20% relative standard error when estimating an indicator with a value of 20% at the first domain. If a particular SPA uses a larger sample than implied by these parameters, the anticipated effect of facility sampling would be less than found in this analysis. Similarly, if a particular SPA uses a smaller sample, the anticipated effect of facility sampling would be larger than found in this study. Nevertheless, SPA samples are not designed to be representative at low levels of disaggregation. Our findings show that this sampling will induce non-trivial errors when linking SPA data from facility samples to DHS clusters.

Another setting constraint was the focus on rural areas as defined by the RIDHS. The health service environment in urban areas is likely to be very different due to the greater density of facilities, a different mix of public and private sector resources, and more transportation options to reach facilities further away. More research is needed on the appropriate way to define the health service environment in urban areas and how to link to relevant populations in a meaningful way.

In this analysis, we attempted to minimize any temporal differences in service environment by selecting two surveys conducted within an 18 month window. However, some measurements of the service environment, such as availability of contraceptive methods, may change rapidly such that additional measurement error may be introduced even when linking surveys that are relatively close together.

Some limitations to the geographic data should be noted. First, no topographic features were considered in this analysis; mountains and forests may naturally impede access to facilities, particularly in Rwanda. Second, although we relied on nationally recognized administrative boundary and road network files, we could not independently verify geographic accuracy of these files. Third, the RSPA facility census excluded small private facilities and the GPS locations were missing for 14 facilities which were thus excluded from the analysis; 12 of these facilities were private. The effect of these exclusions is assumed to be minimal, however, because most private facilities in Rwanda are in or near urban centers. Lastly, SPA GPS data collected prior to 2010 are not publicly available; hence it is not possible to apply these methods to older datasets. The data limitations noted do not detract from this demonstration but may be relevant for analyses that seek to relate service environment with health behaviors and outcomes in other countries.

## Conclusions

The main conclusion from this analysis is that at low levels of geographic disaggregation, we do not recommend linking DHS data to SPA data that are based on independent facility samples. Linking SPA data from a facility census with DHS data at the cluster level is possible for descriptive analyses, but measurement errors associated with geographic displacement of DHS clusters will bias relationships between the service environment and health outcomes. Alternative approaches to collecting detailed facility data that can be linked to DHS or other household survey data have pros and cons. The ability to link facility data to population-based data is one of a number of factors that have to be considered in the design of a facility survey and the extent to which facility surveys can be designed to link with population-based data will depend on the relative priority of these various considerations.

## Abbreviations

ART: Antiretroviral therapy; ARV: Antiretroviral prophylaxis; DHS: Demographic and health survey; EA: enumeration area; FP: Family planning; GPS: Global positioning system; KDE: Kernel density estimation; km: Kilometer; PMTCT: Prevention of mother-to-child transmission; PSU: Primary sampling unit; RIDHS: Rwanda interim demographic and health survey; RSPA: Rwanda service provision assessment; SPA: Service provision assessment; USAID: United States Agency for International Development; VCT: Voluntary testing and counseling; WHO: World Health Organization.

## Competing interests

MPS, SLC, and JS are employed by the University of North Carolina, Chapel Hill, working primarily on the USAID-funded MEASURE Evaluation project. CRB is employed by ICF International working primarily on the USAID-funded MEASURE DHS project. The MEASURE DHS project is responsible for the data collection of the RIDHS and RSPA data used in this analysis.

## Authors’ contributions

SLC, MPS, and CRB were equally involved in the conception and design of this project, with important input from JS. CRB and MPS were responsible for data acquisition and all analyses. All authors were responsible for interpretation of data. MPS and SLC drafted the manuscript and CRB and JS critically reviewed and contributed important intellectual content. All authors read and approved the final manuscript.
